# Molecular diversity of HIV-1 in Croatia

**DOI:** 10.1186/1471-2334-14-S4-O5

**Published:** 2014-05-29

**Authors:** Ivana Grgic, Ana Planinic, Lana Gorenec, Adriana Vince, Josip Begovac, Snjezana Zidovec Lepej

**Affiliations:** 1 Department of Molecular Diagnostics and Flow Cytometry, University Hospital for Infectious Diseases “Dr. Fran Mihaljevic“, Zagreb, Croatia; 2 University of Zagreb, School of Medicine, Zagreb, Croatia

## 

Croatia is a small southeastern European country with a low prevalence of HIV infection and a centralized system of clinical care. The cumulative number of HIV infections in the period 1985-2013 was 1,102. The aim of this study was to analyze the distribution of HIV-1 subtypes in Croatia.

The study enrolled 356 HIV-infected patients (86.2% males) receiving clinical care at the Croatian Reference Center for HIV/AIDS in the period 2000-2013. The patients were classified into three groups based on the year of genotyping and entrance to clinical care (2000-2005, 2006-2010 and 2011-2013). Population-based sequencing of the part of pol gene was performed by using TRUGENE® HIV-1 Genotyping System. HIV-1 subtypes were determined by HIV REGA Subtyping Tool.

Subtype B was detected in 279 of 356 patients (78.37%). In the period 2000-2005, a total of 28.3% (47/166) of patients were infected with non-B subtypes. More recently, percentages of non-B subtypes decreased to 14.9% (20/134 patients, 2006-2010) and 17.8% (10 of 56 patients, 2011-2013). Non-B subtypes were detected in 27 of 49 (55.1%) HIV-infected women. A total of 93.1% (175/188) men who have sex with men (MSM) patients were infected with subtype B.

The majority of HIV-1 infections in Croatia can be attributed to subtype B, particularly among MSM. Non-B subtypes are associated mainly with heterosexual transmission.

